# Anesthetic dreaming, anesthesia awareness and patient satisfaction after deep sedation with propofol target controlled infusion: A prospective cohort study of patients undergoing day case breast surgery

**DOI:** 10.18632/oncotarget.17238

**Published:** 2017-04-19

**Authors:** Marco Cascella, Roberta Fusco, Domenico Caliendo, Vincenza Granata, Domenico Carbone, Maria Rosaria Muzio, Giuseppe Laurelli, Stefano Greggi, Francesca Falcone, Cira Antonietta Forte, Arturo Cuomo

**Affiliations:** ^1^ Department of Anesthesia, Endoscopy and Cardiology, Istituto Nazionale Tumori, IRCCS, Fondazione G. Pascale, Napoli, Italia; ^2^ Department of Diagnostic Imaging, Radiant and Metabolic Therapy, Istituto Nazionale Tumori, IRCCS, Fondazione G. Pascale, Napoli, Italia; ^3^ Department of Emergency Medicine, Umberto I Hospital, Nocera Inferiore, Salerno, Italia; ^4^ Division of Infantile Neuropsychiatry, UOMI, Maternal and Infant Health, Torre del Greco, Napoli, Italia; ^5^ Gynecologic Oncology Surgery, Istituto Nazionale Tumori, IRCCS, Fondazione G. Pascale, Napoli, Italia; ^6^ Psychology, Division of Pain Medicine, Istituto Nazionale Tumori, IRCCS, Fondazione G. Pascale, Napoli, Italia

**Keywords:** anesthesia, anesthesia awareness, deep, intravenous, sedation

## Abstract

**Background:**

Anesthetic dreaming and anesthesia awareness are well distinct phenomena. Although the incidence of intraoperative awareness is more common among patients who reported a dream after surgery, the exact correlation between the two phenomena remains an unsolved rebus. The main purpose of this study was to investigate anesthetic dreaming, anesthesia awareness and psychological consequences eventually occurred under deep sedation. Intraoperative dreaming experiences were correlated with dream features in natural sleep.

**Methods:**

Fifty-one patients, undergoing surgical excision of fibroadenomas under a Bispectral index-guided deep sedation anesthesia with propofol target controlled infusion, were enrolled into this prospective study. Psychological assessment was performed through the State Trait Anxiety Inventory. A questionnaire was adopted to register dreaming and anesthesia awareness. Data were collected after emergence (t0), 24 hours (t1), 1 month (t2), 6 months (t3).

**Results:**

Six patients (12%) reported anesthetic dreaming at t0 confirming the response at each subsequent evaluation. One patient (2%) confirmed dreaming during anesthesia in all, but denied it at t0. There was a high correlation between the intraoperative dream contents and the features of dreams in natural sleep. No cases of anesthesia awareness were detected. A similar level of satisfaction was observed in dreaming and no-dreaming patients.

**Conclusions:**

Anesthetic dreaming does not seem to influence satisfaction of patients undergoing deep sedation with propofol target controlled infusion. A psychological assessment would seem to improve the evaluation of possible psychological consequences in dreamer patient.

## INTRODUCTION

Dreaming during sedation or anesthesia is a fascinating phenomenon and very difficult to study. Similarly to what happens during natural sleep, probably only a limited number of dreams are recalled among all intraoperative experiences [1, 2]. According to Mashour [3], in this discrepancy between dreaming and dream reporting, sedation could represent an optimal test to investigate on the intraoperative dreams because, compared to general anesthesia, this state is more readily reversible and the dreaming subject can be earlier interviewed. Consequently, after sedation the patient could better recall her/his dream. On the other hand, a careful analysis of the dreaming is often complicated by several confounding factors, such as the administration of drugs with amnesic proprieties (e.g., benzodiazepines), or the lack of precise system for the delivery of the intravenous anesthetics. Thus, sedation performed with a general anesthetic alone, and administered with a target controlled infusion (TCI) modality (which enables the drug concentration in the blood or plasma, or at the effect site to be controlled continuously) under a depth of anesthesia (DOA) monitoring guide, could represent an interesting model for research on the relationship between anesthesia and dreams.

The occurrence of dreaming during anesthesia is a well-known phenomenon. In one among the numerous Leslie’s studies on the topic, dreaming was reported in 22% of patients undergoing elective surgery [4], whereas other authors reported an incidence up to 57% [5, 6]. This phenomenon was more evident with ketamine (‘ketamine dreams’) rather than other agents (e.g., propofol) [7]. When ketamine is not used, recent reports showed that intraoperative dreams seems to have similar incidence independent of the anesthetic technique [8]. Previous studies, however, have reported that patients receiving propofol anesthesia had a higher incidence of dreaming than those receiving volatile anesthesia [9, 10]. Concerning dreaming during sedation, two different studies found that approximately 20% to 25% of patients had dreams [11, 12]; another report concluded that the incidence of dreaming was significantly higher during short-term sedation with sevoflurane compared with propofol [13].

Another question concerns the influence of dreams during general anesthesia, or sedation, on the subsequent patient satisfaction (PS). It seems that despite the high frequency of dreaming, this phenomenon does not influence satisfaction or anxiety after anesthesia [5]. Furthermore, in patients recalling dreams the satisfaction was not influenced by the choice of sedative [13].

What is the linkage between anesthetic dreams and the anesthesia awareness with recall (AAWR) phenomenon? This latter is an anesthesia complication consisting in the explicit recall of sensory perceptions of the patient during anesthesia [14]. According to recent data the incidence of certain/probable and possible accidental AAWR cases is around 1:19,600 [15]. In a large cohort of cancer patients we detected an incidence of AAWR of 1:10,550 [16]. While AAWR occurs in extremely rare cases [17-19], patients could experience this complication with procedures that do not necessarily involve general anesthesia, but performed under a different degree of sedation. According to the American Society of Anesthesiology (ASA), in these clinical settings there would be an advantage in brain-function monitoring, especially when used in association with clinical and standard instrumental monitoring of the anesthesia [20].

Although dreaming under anesthesia and AAWRs are well distinct phenomena, the incidence of intraoperative awareness is reported more commonly among patients experienced a dream after surgery. To date, the exact correlation between the two phenomena is an unsolved rebus [21].

The primary aims of this study were to evaluate the incidence and the features of dreaming, and the occurrence of AAWR episodes, during propofol TCI deep sedation conducted under BIS guide. The secondary end points were the evaluation of the PS in dreaming and no-dreaming patients under this type of procedure and the evaluation of possible long-term psychological complications. Dreaming experiences under procedure were correlated with dream features in natural sleep.

## RESULTS

Demographic and intraoperative characteristics, and surgical data, were given in Table 1. Six patients (12%) reported dreaming during anesthesia immediately after the end of the procedure. One patient denied it at the emergence (t0), although she reported a dreaming under sedation after 1 day (t1). She also confirmed it at each subsequent follow-up. Thus, we collected 7 dreaming patients (14%) and 44 non dreaming patients.

**Table 1:** Demographic, Intraoperative and surgical data (n=51)

***Age (years)***	29.35 ± 9.24
***Weight (Kg)***	64.72 ± 11.28
***Height (cm)***	163.30 ± 6.84
***Duration of surgery (minutes)***	24.31 ± 5.27
***Duration of sedation (minutes)***	48.60 ± 9.28
***STAI-State***	34.1 ± 8.8
***STAI-Trait***	30.3 ± 8.3

Data are expressed as mean ± SD

There was a high correlation of the intraoperative dream content with the features of dreams in natural sleep (43%) (Table 2). This correspondence was non obtained in two cases in which patients did not remember the intraoperative dream content in all time observations, as well as in other two patients who usually never remembered their dreams in natural sleep. There were no correlation between dreaming and preoperative acute (State Trait Anxiety Inventory-S, STAI-S), and background (State Trait Anxiety Inventory-T, STAI-T) anxiety, although both patients reporting dreams with no content showed a STAI-S value slightly over the threshold of 40 (Table 2).

**Table 2:** Dream content and correlation with preoperative acute (STAI-S) and background (STAI-T) anxiety (*n=7*)

*Patient n.*	*Dreams description*	*Correspondence with dreams in natural sleep*	*STAI-S/ STAI-T*
**7**	The patient was swimming	She usually never remember her dreams	36/32
**14**	Nice dreaming about her partner	Yes	33/26
**16**	Travelling to somewhere with family	Yes	38/37
**32**	Dream with no content	N/A	42/36
**37**	Imaginary experience of the operation*	She usually never remember her dreams	35/31
**41**	Dream with no content	N/A	41/28
**45**	People from different parts of her life	Yes	34/29

*Although not reported at the emergence, this dream was described in all further follow-up, and with the same features. A careful analysis excluded the occurrence of an anesthesia awareness event

Two patient responded YES to the question “*Can you recall anything in between going to sleep and waking up?*” at the emergence (t0), although after a careful analysis there were no events interpreted as certain episodes of AAWR.

**Figure 1:**
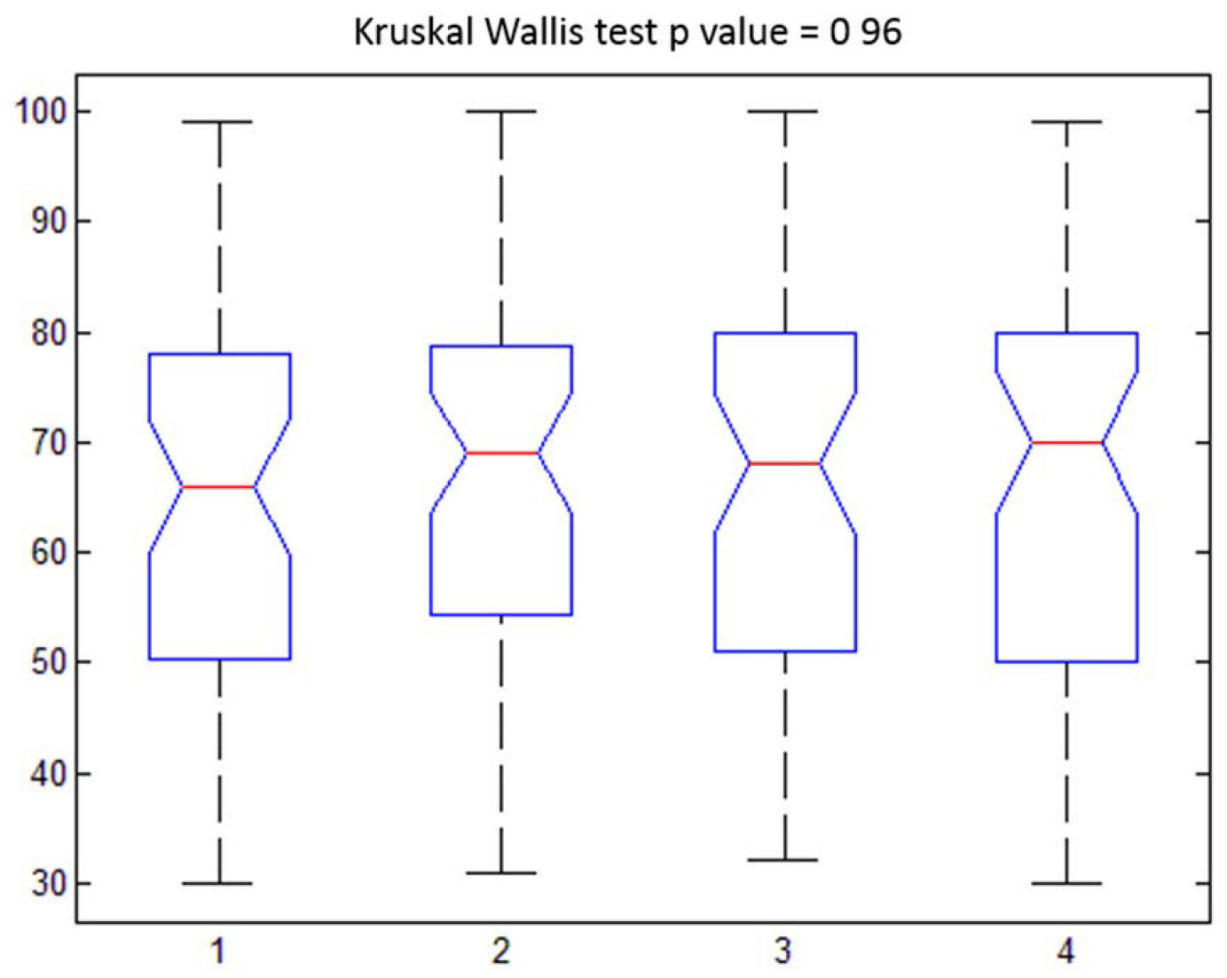
Patient satisfaction (0-100). Boxplot for different time observations with relative p value at Kruskal Wallis test (Clusters: 1=t0; 2=t1; 3=t2; 4=t3)

**Figure 2:**
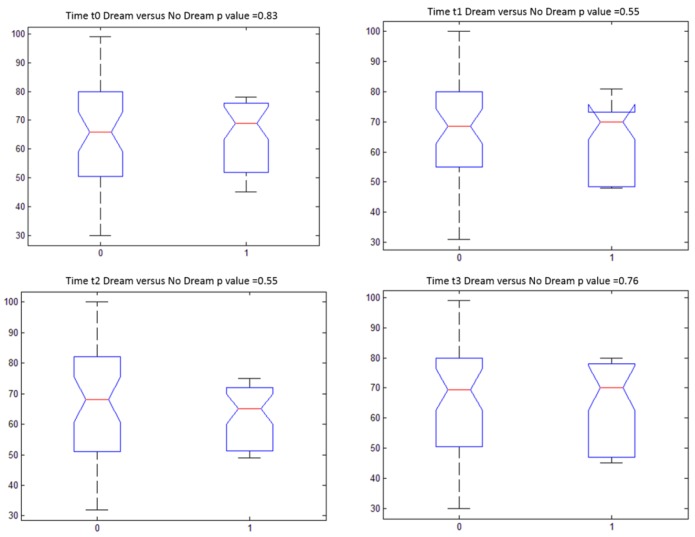
Boxplot of patient satisfaction (0-100) of dream subgroup dream subgroup versus no dream subgroup for each time with relative p value at Kruskal Wallis test (Clusters: 0=Dream subgroup; 1=No Dream subgroup)

About the results of the analysis of the satisfaction scores no significant statistical difference (p-value >0.05 at Kruskal Wallis test) was reported between different time observations (Figure 1). Table 3 shows descriptive statistics of PS for different time observations. Moreover, there was no statistical difference (p-value >0.05 at Kruskal Wallis test) between dreaming and non-dreaming patients with respect to the reported PS. Indeed, a similarly high level of satisfaction was observed in dreaming and no-dreaming patients for each time (Figure 2).

**Table 3:** Descriptive statistics for different time observations of PS (0-100)

	*Min*	*Max*	*Mean*	*Standard Deviation*
***t0***	30	99	66	18.07
***t1***	31	100	69	17.30
***t2***	32	100	68	17.41
***t3***	30	99	70	17.38

PS, patient satisfaction; Emergence (t0); 24 hours (t1); 1 month (t2); 6 months (t3)

## DISCUSSION

This is the first study drawn to evaluate the intraoperative dreaming phenomenon in deep sedation in which Bispectral (BIS) score was used as a guide to titrate propofol TCI. Common methods of monitoring the depth of sedation include the assessment of the patient’s responsiveness, using for instance specific tools, such as the observer’s assessment of awareness/sedation (OAA/S) score. Alternatively, DOA monitors (e.g., BIS monitor) can be adopted, and a recent meta-analysis showed that these tools would impact on sedation titration during interventional procedures with propofol infusions [22]. Several limitations affect DOA monitors and their use remains a major controversy in anesthesiology [23]. On the other side, as well explained by Bagchi et coll. [24], the OAA/S score has the disadvantage of frequent patient stimulation, which may alter the actual level of sedation; in contrast the BIS value would offer a continuous objective assessment with minimal stimulation to patient. Moreover, with this purpose, BIS monitor produces a single number to indicate the level of sedation. According to previous studies, BIS values of 76–85 have been recommended for moderate sedation [25, 26], whereas a higher level of sedation is required for achieving more invasive procedures, such as those included in this study. Therefore, we set the BIS range at 60–75.

In a retrospective analysis Mashour et coll. [27] found that the incidence of AAWR was not statistically different in patients receiving general anesthesia compared with those underwent non-general anesthesia, including sedation. However, data from the Anesthesia Awareness Registry [28] and the 5th National Audit Project (NAP5) [29] underlined that the occurrence of AAWR in patients receiving sedation can be associated with long-term psychological consequences. Because not all AAWR can be detected with interviews or self-reporting, a careful follow-up could be useful to identify postoperative psychological consequences, even when those episodes had been not readily captured at the emergence.

A key problem in all study on the topic regarding the anesthesia awareness is to establish whether the patient’s report can be interpreted as a true episode of AAWR [30, 31]. In lieu of other investigations, vague reports or the description of situations probably occurred in the immediate preoperative or postoperative period (i.e., people talking, application of dressing) were not considered as AAWR events [32]. However, although the anesthesia awareness is often a well-recognizable phenomenon, and several classification instruments (e.g., the Michingan instrument for awareness detection [33]) have been developed, some patients’ reports may result of difficult discernment. The possibility of working in a team in which a psychiatry and a psychologist are present could improve the power of detecting this complication. Consequently, the presence in our investigation of both specialists allowed us to exclude the occurrence of AAWR in a case in which the patient described a potential intraoperative recall after 24 hours from the procedure. This episode occurred in a patient who never remembered her dreams. However, a careful investigation by the psychologist and the psychiatry showed that the features were not related to the actual surgical procedure (i.e., AAWR), but corresponded to a dream where the content was the imaginary experience of the operation. She also confirmed the same description at the further follow-ups (Table 3).

Several structured questions (e.g., ‘Have you observed any changes in the quality of sleep?’, ’Have you recently experienced any flashback?’, ‘Have you noted the appearance of anxiety or panic attack during the day?’, ‘Have you had to resort to taking anti-anxiety drugs or antidepressants after procedure?’, ‘Have you felt changes in your capabilities in the daily work or/and in personal relationships with your family and your friends or in social life?’) are usually used for patient reporting postoperative recall in order to investigate on the psychological sequelae, in acute and long term [34]. After this initial screening, a more detailed psychological assessment is needed in order to establish the triggering and the features of a potential post-traumatic stress disorder (PTSD). This serious condition may develop following a traumatic event and patients who suffered of AAWR have been described as recalling fragments of their surgery in nightmares and flashbacks in which they re-experience paralysis, suffocation, pain, or conversations between surgical personnel [35]. Consequently, these patients usually avoid hospitals, doctors and television programs with hospital themes. Commonly reported hyperarousal symptoms include easy startle, hypervigilance, and irritability [36]. In our study we investigated on the possible occurrence of PTSDs in relation to AAWR and dreaming. Moreover, a psychologist and a psychiatrist - included in our team - explored every possible psychological consequence linked to the procedure, as well as conditions not attributable to PTSD. For instance, in one patient a psychological evaluation showed that sleep disorders (frequent awakenings and nightmares) were attributable to the use of psychotropic substances (see Criterion H of the Diagnostic and Statistical Manual of Mental Disorders-5, DSM-5, diagnostic criteria for PTSD [37]). As result, no subjects met DSM-5 diagnostic criteria for PTSD [37]

The so-called continuity hypothesis states that dreams reflect waking-life experiences [38]. Moreover, many features of dreams reported after procedural sedation were similar to those of natural sleep [39]. In our study, intraoperative dreams showed features of predominantly positive emotions and we found a correlation with the features of dreams in natural sleep in 43% of cases (3/7 dreams). This result is underrated because two dreams under sedation had not content, thus in these two cases the correlation with dreams in natural sleep was not applicable. Further, other two patients who reported an intraoperative dream referred that they usually never remembered their dreams.

The Leslie’s and coauthors study on dreaming after general anesthesia found that dreaming made the patients more depressed and anxious postoperatively, and less satisfied with anesthetic care [40]. This data was not confirmed by our study, because the analysis of PS score between dreaming and no-dreaming patients showed no significant differences. However, compared with the previous Leslie’s investigation, our study presented a different anesthesia setting and it was drawn with a different methodology. Because Gyulahazi et al. [41] demonstrated that dream recalls are more frequent in patients with preoperative suggestions applied before and during induction, in our study patients do not received suggestions. The correlation between dreaming and BIS has been previously investigated. Although studies during general anaesthesia reported that BIS values were not significantly different between dreamers and non-dreamers [42], in the Eer’s study [11] dreaming during sedation was associated with lower BIS values. In our analysis, all patients received deep sedation on the basis of the BIS values. Therefore, BIS was not a variable in the study and BIS did not predict dreams.

### Limitations of the study

This report presents several limitations. Previous studies suggested that sex differences may exist in dreaming under anesthesia and during propofol sedation. In a study conducted in patients under general anesthesia women were more likely to report dreams than men [40], whereas the incidence of dreaming was significantly higher in men than in women during short propofol sedation for upper gastrointestinal endoscopy [43]. However, the investigation on sex differences in dreaming patients was not an objective this reports.

In this study there was not a control group receiving another type of sedation (e.g., midazolam, or propofol and remifentanil). However, our aim was to evaluate the occurrence of dreams in patients underwent a specific protocol for propofol sedation and based on a TCI infusion under BIS guide, rather than demonstrate the different impact on the phenomenon of different drugs or protocols.

Again, we underline that the current study was conducted among patients of similar age, therefore it is not suitable to evaluate the effect of age on dreaming.

Further, a PTSD may develop after months/years after the occurrence of the trauma. Thus, a six-months follow-up might seem a limited observation time. However, because our aim was to evaluate the correlation between dreaming, anesthesia awareness and potential psychological sequelae, six months represent a good window in order to detect an episode of dreaming and/or recall of sensory perceptions during anesthesia, and to correlate it to the occurrence of a stress response.

In summary, in the present study dreaming was observed in 14% of patients undergoing propofol TCI deep sedation, under BIS guide. Moreover, dreams did not influence PS and there was a high correlation of the intraoperative dream content with the features of dreams in natural sleep. It may be necessary to differentiate between dreams and recall of intraoperative events. The psychological assessment helped us to evaluate a possible case of AAWR, as well as to evaluate and manage a possible case of long-term psychological consequence. Further studies are necessary to better investigate on the correlation between dream and anesthesia. Patients during anesthesia must be necessarily unconsciousness, but at the same time through a dream our brain generates a conscious experiences by itself. Consequently, this field of study appears to be closed the scientific research regarding the nature of consciousness, as well as the operating mechanisms of anesthetics and the transition between the consciousness/unconsciousness states under anesthesia.

## MATERIAL AND METHODS

### Study population and design

Fifty-one patients undergoing surgical excision of fibroadenomas under deep sedation anesthesia in our hospital were included in this prospective cohort study from March 2015 to September 2015. Approval from the Institutional Medical Ethical Committee (protocol number 43/14 oss) of the Istituto Nazionale Tumori, Fondazione Pascale, Naples was obtained and the patients gave their informed consent. All procedures were executed in a day case setting, where patients were discharged the same day. Patients refusing to participate in the study, accept brain monitoring or receive sedation during the surgery were excluded. Other exclusion criteria included patients aged less than 18, pregnant patients, and those do not complying to ASA I/ASA II criteria or with history of drug abuse, psychiatric illnesses, cognitive impairments, and those who had taken hypnotics.

### Preoperative psychological assessment and anesthesia management

Preoperative psychological and psychiatric assessment was performed by two specialists in the team in order to identify exclusion criteria, and to measure the psychological stress level. For this purpose we followed the same methodology used by Aceto et associates [44]. The Spielberger’ STAI tool, a questionnaire consisting of two 20-item subscales, one of which measures state anxiety (acute state anxiety, STAI-S) and the other trait anxiety (background trait anxiety, STAI-T), was adopted. Respondents rated 4-point rating scales that were scored from 1 (not at all) through 4 (very much) for the STAI-S and from 1 (almost never) through 4 (almost always) for the STAI-T. The total score ranges 20-80 in each subscale. A threshold of 40 indicates the presence of an anxiety state of an increasing grade, as the score increases [45]. The tests were performed on the day of surgery, approximately 30 min before induction of sedation. No premedication was administered, in order to avoid any confounding effects on memory, and no suggestions were used immediately before the start of the sedation, to not influence the patients’ imagination. The sedation was performed with propofol, administered with a TCI system (Orchestra® Fresenius Kabi) using the Schnider model [46]. A starting effect-site concentration (Ce) of 2,5 ng/ml was established [47]. Assisted ventilation of the lung was performed by a facemask with oxygen-enriched air (O2 30%).

Brain monitoring was obtained using a BIS monitor (BIS VISTA Monitoring System, Covidien, Mansfield, MA, USA). A BIS sensor was applied to the forehead of all patients after skin preparation with alcohol in order to achieve an impedance <5 kΩ. A Signal Quality Index (SQI) >80% was considered appropriate for data registration. The propofol TCI was titrated to maintain the target range of the BIS index between 60 and 75. The SpO2, heart rate (HR), respiratory rate (RR) and BIS were monitored continuously, and the mean arterial pressure (MAP) continually at 5 min intervals, until the end of surgery. After achieving the deep sedation, patients received a pre-incisional wound infiltration with local anesthetic (0.5% bupivacaine, 10 ml).

### Intraoperative experiences evaluation and follow-ups

When the patients initially emerged from sedation and were oriented to the time, place, and person (t0), they were asked the following standardized questions, composed from the original Brice’s questionnaire [48]:

1. “What is the last thing you remember before going to sleep?”

2. “What is the first thing you remember when you woke up?”

3. “Can you recall anything in between going to sleep and waking up?”

4. “Did you have any dreams during your procedure?”

5. “Please assign a score for expressing your satisfaction with the sedation” (0 no satisfaction, 100 maximum satisfaction)

All possible dream narrative reports (question 4) were reviewed by the anesthesiologist and the psychologist to investigate whether the patient’s report represented an explicit recall of sensory perceptions patient during anesthesia or a true dream. When the review of the report allowed us to exclude the occurrence of an episode of AAWR, the dream was included in the analysis and patients (dream subgroup) were asked to indicate the correspondence of the intraoperative dream features with those in dreams of their natural sleep.

The five questions questionnaire was repeated through telephonic interview after 24 hours (t1), 1 months (t2) and 6 months (t3) from surgery. In each observation time (t0, t1, t2, t3) a score for PS, ranging from 0 (no satisfaction) to 100 (maximum satisfaction) (question 5: “*Please assign a score for expressing your satisfaction with the sedation*”), was registered for both dreaming and no dreaming patients (no dream subgroup) in order to establish a possible correlation between dream and PS.

### Statistical analysis

Data were expressed in terms of median value and standard deviation. Kruskal Wallis non-parametric test was performed to emphasize significant statistically difference between median value in different population groups (to, t1, t2, t3 and dream versus no dream groups).

A *p*-value <0.05 was considered statistically significant. All analyses were performed using Statistics Toolbox of Matlab R2007a (The Math-Works Inc., Natick, MA).

### Highlights

1. Although dreaming during anesthesia and sedation is a well-known phenomenon, it seems that this phenomenon does not influence satisfaction or anxiety after anesthesia.

2. There is a high correlation of the intraoperative dream content with the features of dreams in natural sleep.

3. It may be necessary to differentiate between dreams or recall of intraoperative events.

4. A careful psychological assessment may support anesthesiologists in order to detect, evaluate and manage possible cases of long-term psychological consequences after dreaming and/or recalls.

## Abbreviations

TCI: target controlled infusion
DOA: depth of anesthesia
PS: Patient Satisfaction
AAWR: anesthesia awareness with recall
ASA: American Society of Anesthesiology
STAI-S: State Trait Anxiety Inventory-S
STAI-T: State Trait Anxiety Inventory-T
BIS: Bispectral index
OAA/S: observer’s assessment of awareness/sedation
NAP5: 5th National Audit Project
PTSD: post traumatic stress disorder
DSM-5: Diagnostic and Statistical Manual of Mental Disorders-5
Ce: effect-site concentration
SQI: Signal Quality Index
HR: heart rate
RR: respiratory rate
MAP: mean arterial pressure

## References

[1] Rosen MG (2013). What I make up when I wake up: anti-experience views and narrative fabrication of dreams. Front Psychol.

[2] Windt JM (2013). Reporting dream experience: Why (not) to be skeptical about dream reports. Front Hum Neurosci.

[3] Mashour GA (2011). Dreaming during anesthesia and sedation. Anesth Analg.

[4] Leslie K, Skrzypek H, Paech MJ, Kurowski I, Whybrow T (2007). Dreaming during anesthesia and anesthetic depth in elective surgery patients: a prospective cohort study. Anesthesiology.

[5] Hellwagner K, Holzer A, Gustorff B, Schroegendorfer K, Greher M, Weindlmayr-Goettel M, Saletu B, Lackner FX (2003). Recollection of dreams after short general anaesthesia: influence on patient anxiety and satisfaction. Eur J Anaesthesiol.

[6] Samuelsson P, Brudin L, Sandin RH (2008). Intraoperative dreams reported after general anaesthesia are not early interpretations of delayed awareness. Acta Anaesthesiol Scand.

[7] Sarasso S, Boly M, Napolitani M, Gosseries O, Charland-Verville V, Casarotto S, Rosanova M, Casali AG, Brichant JF, Boveroux P, Rex S, Tononi G, Laureys S (2015). Consciousness and Complexity during Unresponsiveness Induced by Propofol, Xenon, and Ketamine. Curr Biol.

[8] Gyulaházi J, Varga K, Iglói E, Redl P, Kormos J, Fülesdi B (2015). The effect of preoperative suggestions on perioperative dreams and dream recalls after administration of different general anesthetic combinations: a randomized trial in maxillofacial surgery. BMC Anesthesiology.

[9] Leslie K, Sleigh J, Paech MJ, Voss L, Lim CW, Sleigh C (2009). Dreaming and electroencephalographic changes during anesthesia maintained with propofol or desflurane. Anesthesiology.

[10] Marsch SCU, Schaefer HG, Tschan C, Meier B (1992). Dreaming and anaesthesia: total i.v. anaesthesia with propofol versus balanced volatile anaesthesia with enflurane. Eur J Anaesthesiol.

[11] Eer AS, Padmanabhan U, Leslie K (2009). Propofol dose and incidence of dreaming during sedation. Eur J Anaesthesiol.

[12] Stait ML, Leslie K, Bailey R (2008). Dreaming and recall during sedation for colonoscopy. Anaesth Intensive Care.

[13] Xu GH, Liu XS, Yu FQ, Gu EW, Zhang J, Royse AG, Wang K (2012). Dreaming during sevoflurane or propofol short-term sedation: a randomised controlled trial. Anaesth Intensive Care.

[14] Mashour GA, Orser BA, Avidan MS (2011). Intraoperative awareness: from neurobiology to clinical practice. Anesthesiology.

[15] Pandit JJ, Andrade J, Bogod DG, Hitchman JM, Jonker WR, Lucas N, and the Royal College of Anaesthetists; Association of Anaesthetists of Great Britain and Ireland (2014). 5^th^ National Audit Project (NAP5) on accidental awareness during general anaesthesia: summary of main findings and risk factors. Br J Anaesth.

[16] Cascella M, Viscardi D, Schiavone V, Mehrabmi-Kermani F, Muzio MR, Forte CA, De Falco F, Barberio D, Cuomo A (2016). A 7-Year Retrospective Multisource Analysis on the Incidence of Anesthesia Awareness With Recall in Cancer Patients: A Chance of Collaboration Between Anesthesiologists and Psycho-Oncologists for Awareness Detection. Medicine (Baltimore).

[17] Cascella M, Bifulco F, Viscardi D, Tracey MC, Carbone D, Cuomo A (2016). Limitation in monitoring depth of anesthesia: a case report. J Anesth.

[18] Ranta SO, Herranen P, Hynynen M (2002). Patients’ conscious recollections from cardiac anesthesia. J Cardiothorac Vasc Anesth.

[19] Chung HS (2014). Awareness and recall during general anesthesia. Korean J Anesthesiol.

[20] American Society of Anesthesiologists Task Force on Intraoperative Awareness (2006). Practice advisory for intraoperative awareness and brain function monitoring: a report by the American society of anesthesiologists task force on intraoperative awareness. Anesthesiology.

[21] Samuelsson P, Brudin L, Sandin RH (2008). Intraoperative dreams reported after general anaesthesia are not early interpretations of delayed awareness. Acta Anaesthesiol Scand.

[22] Conway A, Sutherland J (2016). Depth of anaesthesia monitoring during procedural sedation and analgesia: A systematic review and meta-analysis. Int J Nurs Stud.

[23] Cascella M (2016). Mechanisms underlying brain monitoring during anesthesia: limitations, possible improvements, and perspectives. Korean Journal of Anesthesiology.

[24] Bagchi D, Mandal MC, Das S, Basu SR, Sarkar S, Das J (2013). Bispectral index score and observer’s assessment of awareness/sedation score may manifest divergence during onset of sedation: Study with midazolam and propofol. Indian J Anaesth.

[25] Johansen JW (2006). Update on bispectral index monitoring. Best Practice and Research: Clinical Anaesthesiology.

[26] Bower AL, Ripepi A, Dilger J, Boparai N, Brody FJ, Ponsky JL (2000). Bispectral index monitoring of sedation during endoscopy. Gastrointest Endosc.

[27] Mashour GA, Wang LY, Turner CR, Vandervest JC, Shanks A, Tremper KK (2009). A retrospective study of intraoperative awareness with methodological implications. Anesth Analg.

[28] Kent CD, Mashour GA, Metzger NA, Posner KL, Domino KB (2013). Psychological impact of unexpected explicit recall of events occurring during surgery performed under sedation, regional anaesthesia, and general anaesthesia: data from the Anesthesia Awareness Registry. Br J Anaesth.

[29] Cook TM, Andrade J, Bogod DG, Hitchman JM, Jonker WR, Lucas N, Mackay JH, Nimmo AF, O’Connor K, O’Sullivan EP, Paul RG, Palmer JH, Plaat F (2014). Royal College of Anaesthetists; Association of Anaesthetists of Great Britain and Ireland. 5th National Audit Project (NAP5) on accidental awareness during general anaesthesia: patient experiences, human factors, sedation, consent, and medicolegal issues. Br J Anaesth.

[30] Mashour GA, Avidan MS (2015). Intraoperative awareness: controversies and non-controversies. Br J Anaesth.

[31] Mashour GA, Kent C, Picton P, Ramachandran SK, Tremper KK, Turner CR, Shanks A, Avidan MS (2013). Assessment of intraoperative awareness with explicit recall: a comparison of 2 methods. Anesth Analg.

[32] Sandin RH, Enlund G, Samuelsson P, Lennmarken C (2000). Awareness during anaesthesia: a prospective case study. Lancet.

[33] Mashour GA, Esaki RK, Tremper KK, Glick DB, O’Connor M, Avidan MS (2010). A novel classification instrument for intraoperative awareness events. Anesth Analg.

[34] Osterman JE, Hopper J, Heran WJ, Keane TM, van der Kolk BA (2001). Awareness under anesthesia and the development of posttraumatic stress disorder. Gen Hosp Psychiatry.

[35] Moerman N, Bonke B, Oosting J (1993). Awareness and recall during general anesthesia. Anesthesiology.

[36] Osterman JE, van der Kolk BA (1998). Awareness during anesthesia and posttraumatic stress disorder. Gen Hosp Psychiatry.

[37] Friedman MJ, Resick PA, Bryant RA, Strain J, Horowitz M, Spiegel D (2011). Classification of trauma and stressor-related disorders in DSM-5. Depress Anxiety.

[38] Schredl M (2010). Characteristics and contents of dreams. Int Rev Neurobiol.

[39] Stait ML, Leslie K, Bailey R (2008). Reaming and recall during sedation for colonoscopy. Anesth Intensive Care.

[40] Leslie K, Myles P, Forbes A, Chan MT, Swallow SK, Short TG (2005). Dreaming during anaesthesia in patients at high risk of awareness. Anaesthesia.

[41] Gyulahazi J, Varga K, Igloi E, Redl P, Kormos J, Fulesdi B (2015). The effect of preoperative suggestions on perioperative dreams and dream recalls after administration of different general anesthetic combinations: a randomized trial in maxillofacial surgery. BMC Anesthesiol.

[42] Samuelsson P, Brudin L, Sandin RH (2008). BIS does not predict dreams reported after anaesthesia. Acta Anaesthesiol Scand.

[43] Xu G, Liu X, Sheng Q, Yu F, Wang K (2013). Sex differences in dreaming during short propofol sedation for upper gastrointestinal endoscopy. Neuroreport.

[44] Aceto P, Perilli V, Lai C, Sacco T, Modesti C, Luca E, De Santis P, Sollazzi L, Antonelli M (2015). Minimum alveolar concentration threshold of sevoflurane for postoperative dream recall. Minerva Anestesiol.

[45] Spielberger CD, Gorsuch RL, Lushene R, Vagg PR, Jacobs GA (1983). Manual for the State-Trait Anxiety Inventory (Form Y).

[46] Schnider TW, Minto CF, Shafer SL, Gambus PL, Andresen C, Goodale DB, Youngs EJ (1999). The influence of age on propofol pharmacodynamics. Anesthesiology.

[47] Hong J-Y, Jee YS, Luthardt FW (2005). Comparison of conscious sedation for oocyte retrieval between low-anxiety and high-anxiety patients. J Clin Anesth.

[48] Brice DD, Hetherington RR, Utting JE (1970). A simple study of awareness and dreaming during anaesthesia. Br J Anaesth.

